# Creatine to Augment Bioenergetics in Alzheimer's Disease

**DOI:** 10.1002/alz70858_100477

**Published:** 2025-12-24

**Authors:** Matthew K Taylor

**Affiliations:** ^1^ University of Kansas Medical Center, Kansas City, KS, USA

## Abstract

**Background:**

Brain metabolism, including the creatine (Cr) system, is impaired in Alzheimer's disease (AD). Creatine monohydrate (CrM) supplementation is suggested to improve AD pathophysiology and symptoms in AD mouse models. No human studies have been reported to date; thus, we investigated the feasibility of eight‐week CrM supplementation in AD and generated preliminary evidence for its potential effects on bioenergetics and cognition.

**Method:**

Twenty participants with dementia due to probable AD were allocated to a single‐arm, open‐label, eight‐week intervention of 20 g/day CrM. Compliance was tracked daily via participant self‐reports (with assistance from study partners). At baseline, 4‐, and 8‐week visits, we measured fasting serum Cr. At baseline and 8‐week visits, we measured whole brain total Cr (as a ratio to unsuppressed water) using ^1^H magnetic resonance spectroscopy (MRS), lymphocyte ADP and ATP production, platelet and lymphocyte mitochondrial respiration, and cognition using the NIH Toolbox Cognition Battery. We report descriptive feasibility and used paired t‐tests to test for changes from baseline in outcomes biomarkers. Statistical significance was set at *p* <0.05.

**Result:**

Participants had a mean age of 73.1 ± 6.3 years and were 65% male. No participants withdrew from the study and 95% self‐reported exceeding the compliance target of consuming ≥80% of expected CrM doses. Compared to baseline, serum Cr levels increased 8.4‐fold at 4 weeks and 9.0‐fold at 8 weeks (*p* <0.001). Whole brain total Cr increased by 11.0% (*p* <0.001). Lymphocyte ATP (2.9‐fold, *p* = 0.004) and ADP (2.3‐fold, *p* = 0.02) production increased. Among the entire sample, platelet mitochondrial respiration did not change; yet, in females, State 2 (3.7‐fold, *p* = 0.04), State 3 (3.5‐fold, *p* = 0.004), and Maximum (2.9‐fold, *p* = 0.05) respiration increased. Cognition improved by 4.4 points in global fluid cognition (*p* = 0.004), 8 points in working memory (*p* <0.001), and 5 points in executive function (*p* = 0.05).

**Conclusion:**

This pilot trial demonstrated that acute intervention with 20 g/day of CrM is feasible in patients with AD and associated with bioenergetic and cognitive improvements. These preliminary data suggest further investigation into CrM's brain target engagement and potential as an affordable adjuvant therapy is justifiable.

